# The Role of Imaging Techniques in the Evaluation of Extraglandular Manifestations in Patients with Sjögren’s Syndrome

**DOI:** 10.3390/diagnostics16020358

**Published:** 2026-01-22

**Authors:** Marcela Iojiban, Bogdan-Ioan Stanciu, Laura Damian, Lavinia Manuela Lenghel, Carolina Solomon, Monica Lupșor-Platon

**Affiliations:** 1 Department of Radiology and Imaging. Nuclear Medicine, “Iuliu Hațieganu” University of Medicine and Pharmacy, 400162 Cluj-Napoca, Romania; mmarcela1612@gmail.com (M.I.); stanciu.bogdanioan96@gmail.com (B.-I.S.); monica.lupsor@umfcluj.ro (M.L.-P.); 2 Department of Rheumatology, Cluj County Emergency Clinical Hospital, 400006 Cluj-Napoca, Romania; ldamian.reumatologie@gmail.com; 3 Department of Radiology and Medical Imaging, Cluj County Emergency Clinical Hospital, 400347 Cluj-Napoca, Romania; lenghel.manuela@gmail.com; 4 Department of Radiology and Imaging, “Iuliu Hațieganu” University of Medicine and Pharmacy, 400012 Cluj-Napoca, Romania; 5 Department of Medical Imaging, “Prof. Dr. Octavian Fodor” Regional Institute of Gastroenterology and Hepatology, 400394 Cluj-Napoca, Romania

**Keywords:** Sjögren’s syndrome, extraglandular manifestations, inclusion body myositis, synovitis, interstitial lung disease, neurological complications, MALT lymphoma, ultrasonography, elastography, high-resolution computed tomography, magnetic resonance imaging

## Abstract

Sjögren’s syndrome is a chronic autoimmune disease marked by lymphocytic infiltration of the exocrine glands and the development of sicca symptoms, yet some patients also develop extraglandular involvement. Imaging has become relevant for describing these systemic features and supporting clinical assessment. This review discusses the roles of ultrasonography, elastography, computed tomography, and magnetic resonance imaging in evaluating multisystem disease associated with Sjögren’s syndrome. Ultrasonography and elastography help assess muscular involvement by showing changes in echogenicity and stiffness that reflect inflammation and later tissue remodeling. In joints, ultrasound can detect synovitis, tenosynovitis, and early erosive changes, including abnormalities not yet evident on examination. Pulmonary disease, most often with interstitial lung involvement, is best evaluated with high-resolution computed tomography, which remains the most reliable imaging modality for distinguishing interstitial patterns. Magnetic resonance imaging is valuable in assessing neurological complications. It can reveal ischemic and demyelinating lesions, neuromyelitis optica spectrum features, or pseudotumoral appearances. Imaging is also essential for detecting lymphoproliferative complications, for which ultrasound and magnetic resonance imaging can reveal characteristic structural and diffusion-weighted imaging findings. When combined with clinical and laboratory information, these imaging methods improve early recognition of systemic involvement and support accurate monitoring of disease progression in Sjögren’s syndrome.

## 1. Introduction

Sjögren’s syndrome is a multisystem autoimmune disease with a chronic course, characterized by lymphocytic infiltration of the exocrine glands, particularly the salivary and lacrimal glands, and clinically reflected by the development of sicca syndrome [1,2,3,4,5,6]. In addition to salivary gland involvement, several organ systems may also be affected in Sjögren’s syndrome [3,4,5].

Sjögren’s syndrome affects approximately 0.5–1% of the general population and shows a marked predilection for the female sex, with an estimated female-to-male ratio of nine to one. The average age at diagnosis is around 50 years [2].

The diagnosis of Sjögren’s syndrome is made according to the classification criteria established by ACR/EULAR (American College of Rheumatology/European League Against Rheumatism, currently renamed the European Alliance of Associations for Rheumatology), published in 2016 [7,8].

Modern imaging techniques, such as ultrasonography (US), elastography (SE/SWE), computed tomography (CT), and magnetic resonance imaging (MRI), allow both the characterization of glandular involvement by detecting structural changes in the salivary glands and the evaluation of systemic involvement (muscular and articular, pulmonary, neurological, and lymphoproliferative complications), with the advantage of avoiding the invasive nature of traditional methods and reducing radiation exposure [8,9,10,11,12,13,14,15,16,17].

This paper aims to analyze the role of imaging techniques in evaluating systemic involvement, including the muscular, articular, pulmonary, and neurological systems, as well as lymphoma development, in patients with Sjögren’s syndrome.

## 2. Methods

This narrative review was based on a focused literature search conducted in PubMed/MEDLINE to identify publications addressing the role of imaging in systemic (extraglandular) manifestations of Sjögren’s syndrome. The search combined Medical Subject Headings (MeSH) and free-text terms related to Sjögren’s syndrome with imaging modalities, including ultrasonography, elastography, computed tomography/high-resolution computed tomography, and magnetic resonance imaging, as well as organ-specific terms referring to musculoskeletal, pulmonary, neurological, and lymphoproliferative involvement. Additional relevant articles were identified through manual screening of reference lists from selected publications. The retrieved evidence was qualitatively analyzed and synthesized, and the results are presented in a narrative format.

All diagnostic imaging figures (ultrasound, elastography, computed tomography, and magnetic resonance imaging) are original and belong to the authors; they were acquired during routine clinical care and fully anonymized before inclusion. The images are presented for illustrative purposes in this narrative review.

## 3. Imaging Evaluation of Systemic Manifestations in Sjögren’s Syndrome

Sjögren’s syndrome may present with either glandular manifestations (sicca syndrome) or systemic, extraglandular manifestations (Table 1) [18,19]. In a multicenter study, Seror and colleagues reported that, among 395 patients with primary Sjögren’s syndrome, 30% presented systemic manifestations at the time of evaluation, and 39% had previously experienced extraglandular symptoms [20].

**Table 1:** Reported prevalence of systemic manifestations in primary Sjögren’s syndrome [21,22,23,24,25].

Systemic Manifestations	Prevalence (%)
Muscle involvement	<2
Joint involvement	53
Pulmonary involvement	23
Central nervous system involvement	10.8

EULAR developed the ESSDAI score (EULAR Sjögren’s Syndrome Disease Activity Index) to quantify systemic involvement in Sjögren’s syndrome, and it serves as a standardized tool for assessing disease activity across 12 domains: constitutional (non-specific symptoms such as fever, night sweats, and unintentional weight loss), glandular, articular, muscular, pulmonary, renal, central and peripheral neurological, lymphoma/lymphadenopathy, cutaneous, hematological, and biological [19]. Several of these domains, such as the articular, muscular, pulmonary, neurological, and lymphoma domains, can be assessed by imaging, providing additional information to evaluate the severity of systemic involvement (Table 2).

**Table 2:** Imaging techniques used for the evaluation of systemic manifestations in Sjögren’s syndrome.

Systemic Involvement	Findings	Imaging Technique
Muscular	Myositis	Ultrasonography Elastography
Articular	Synovitis RC ^1^ joint MCP ^2^ joint PIP ^3^ joint Tenosynovitis Erosive arthritis	Ultrasonography
Pulmonary	Interstitial lung disease	High-resolution computed tomography
Central nervous system	Demyelinating lesions Neuromyelitis optica Pseudotumoral brain lesions	Magnetic resonance imaging
Lymphoproliferative	Lymphoma	Ultrasonography Magnetic resonance imaging

^1^ RC = radiocarpal; ^2^ MCP = metacarpophalangeal; ^3^ PIP = proximal interphalangeal.

### 3.1. Imaging Evaluation of Muscle Involvement in Sjögren’s Syndrome

Histopathologically confirmed myositis is a rare manifestation in Sjögren’s syndrome (<2%) [21,22], characterized by various histological patterns within the spectrum of idiopathic inflammatory myopathies, such as inclusion body myositis, polymyositis, and dermatomyositis [26]. Espitia-Thibault et al. identified a non-specific form of myositis associated with Sjögren’s syndrome, characterized by follicular organization of the lymphocytic infiltrate, suggesting a shared pathogenesis between muscular and glandular involvement in the context of the autoimmune disease [27].

Inclusion body myositis is the most frequent form of myositis associated with Sjögren’s syndrome [26,28]. Imaging techniques are commonly used in clinical practice for patients presenting with muscle weakness, as in idiopathic inflammatory myopathies [29,30,31].

Ultrasonography is a valuable tool for evaluating structural changes in myositis and can be used both for diagnostic purposes and for monitoring disease progression [31]. Ultrasonographic examination shows increased muscle echogenicity in the myositis subtypes mentioned above, along with reduced muscle thickness, the reduction being significantly greater in inclusion body myositis than in other idiopathic inflammatory myopathies or in healthy subjects [29]. The increase in echogenicity results from inflammatory infiltration in the acute phase, during which the bone cortex remains well visualized, while in the chronic phase, it is predominantly due to fatty infiltration, fibrosis, and muscle atrophy [32]. Ultrasonography has a sensitivity and specificity of 82% and 98%, respectively, for the diagnosis of inclusion body myositis [33]. Muscle echogenicity can be assessed ultrasonographically either quantitatively, by placing a region of interest within the examined muscle and measuring its gray-scale value, or semi-quantitatively using the Heckmatt scale (Table 3) [34].

**Table 3:** The Heckmatt scale for evaluating muscle echogenicity [34].

Grade	Clinical Significance	Ultrasonographic Appearance
1	Normal	Hypoechoic muscle with a clearly visible bone cortex
2	Mild changes	Mild increase in muscle echogenicity, with a clearly visible bone cortex
3	Moderate changes	Marked increase in muscle echogenicity, with blurring of the bone cortex
4	Severe changes	Markedly hyperechoic muscle, with no visualization of the bone cortex

**Figure 1:**
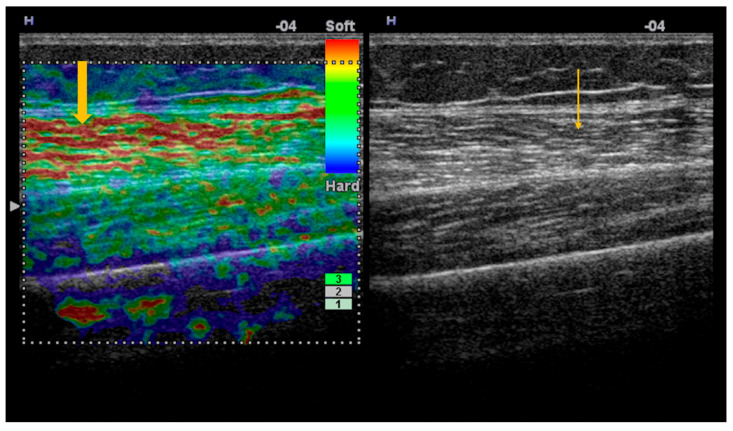
Strain elastography and corresponding gray-scale imaging of myositis with fatty infiltration. Strain elastography of the thigh skeletal muscle demonstrates reduced tissue stiffness, as indicated by red color coding on the elastography map (thick arrow), indicating softening of the affected muscle. The corresponding gray-scale ultrasound image shows hyperechoic muscle texture (thin arrow), consistent with fatty infiltration.

Elastography, both strain and shear-wave, provides additional information to conventional ultrasonography, demonstrating increased stiffness in the acute phase of myositis and reduced stiffness during disease progression, in the context of fatty infiltration (Figure 1) [35]. Because standardized reference values for normal muscle stiffness are not available in the literature, studies rely on comparisons between patients with Sjögren’s syndrome and healthy subjects. In patients with inclusion body myositis, muscle stiffness measured by shear-wave elastography (m/s) is lower compared with normal muscle: vastus lateralis (1.35 ± 0.32 vs. 1.68 ± 0.23), rectus femoris (1.52 ± 0.33 vs. 1.81 ± 0.23), vastus medialis (1.36 ± 0.16 vs. 1.60 ± 0.21) vastus intermedius (1.62 ± 0.49 vs. 1.86 ± 0.22), biceps femoris (1.30 ± 0.14 vs. 1.67 ± 0.20), semitendinosus (1.33 ± 0.31 vs. 1.66 ± 0.23), semimembranosus (1.36 ± 0.28 vs. 1.71 ± 0.18) (*p* < 0.05) [36].

### 3.2. Imaging Evaluation of Joint Involvement in Sjögren’s Syndrome

The joints are frequently involved in the systemic inflammatory process of Sjögren’s syndrome, being affected in approximately 53% of patients [23]. Clinical manifestations range from arthralgia without objective inflammatory signs in the joints to inflammatory arthritis (16%)—most often non-erosive, or more rarely erosive, similar to that observed in rheumatoid arthritis (5%) [37,38]. Articular involvement is usually symmetric and affects the radiocarpal (RC), metacarpophalangeal (MCP), and proximal interphalangeal (PIP) joints (Table 4) [37].

**Table 4:** Reported prevalence of joint involvement by anatomical site in primary Sjögren’s syndrome [4].

Joints	Prevalence (%)
Radiocarpal	30
Metacarpophalangeal	35
Proximal interphalangeal	35

Ultrasonography is a valuable imaging tool for identifying articular changes in Sjögren’s syndrome, such as synovitis, tenosynovitis, or bone erosions, including in cases with subclinical involvement that cannot be detected on physical examination [38,39]. Ultrasound of the small joints of the hand (RC, MCP, and PIP) has a sensitivity of 73%, 64%, and 71%, and a specificity of 78%, 93%, and 94% for the diagnosis of synovitis, with an area under the curve of 0.81, 0.91, and 0.91, respectively [40].

**Figure 2:**
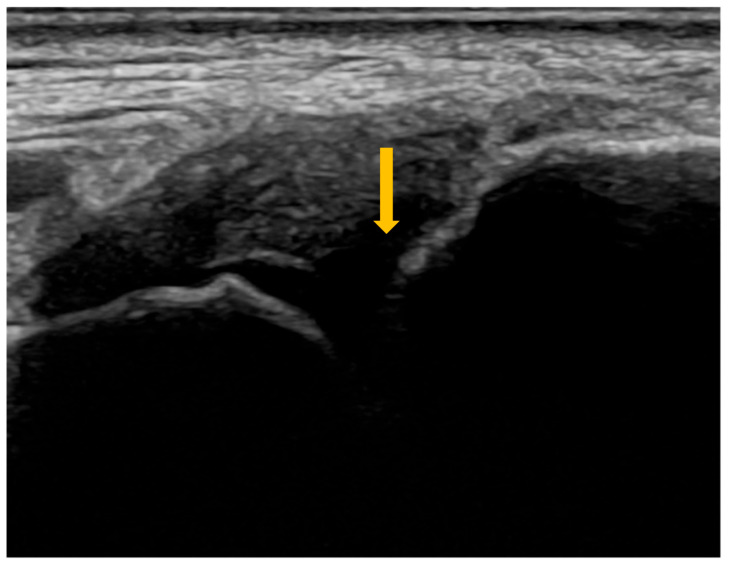
B-mode ultrasound of the right radiocarpal joint demonstrating synovial hypertrophy. Gray-scale (B-mode) ultrasound of the right radiocarpal joint shows synovial hypertrophy, appearing as thickened hypoechoic synovial tissue within the joint recess (arrow), consistent with active inflammation.

On ultrasound, synovitis is characterized by synovial hypertrophy (Figure 2) and joint effusion, with an increased-color Doppler signal suggestive of vascular congestion [38]. The severity of synovitis is assessed using a semi-quantitative scoring system developed by the OMERACT group (Outcome Measures in Rheumatology), which allows classification of patients into different categories of inflammatory activity (Table 5) [41].

**Table 5:** The OMERACT score for classifying synovitis [41].

Grade	Synovitis	Synovial Hypertrophy on B-Mode US	Power Doppler Signal	Combined Score
0	Absent	No SH ^1^, regardless of effusion	No PD ^2^	No SH ^1^ or PD ^2^
1	Minimal	SH ^1^ not extending beyond the horizontal line connecting the bony surfaces, regardless of effusion	≤3 isolated spots ≤1 confluent spot + 2 isolated spots ≤2 confluent spots	SH ^1^ Grade 1 and PD ^2^ ≤ Grade 1
2	Moderate	SH ^1^ extending beyond the joint line, with a linear or concave surface, regardless of effusion	>Grade 1, with PD ^2^ spots < 50% of the SH ^1^ area	SH ^1^ Grade 2 and PD ^2^ ≤ Grade 2 OR SH ^1^ Grade 1 and PD ^2^ Grade 2
3	Severe	SH ^1^ extending beyond the joint line, with a convex surface, regardless of effusion	>Grade 2, with PD ^2^ spots > 50% of the SH ^1^ area	SH ^1^ Grade 3 and PD ^2^ ≤ Grade 3 OR SH ^1^ Grade 1 or 2 and PD ^2^ Grade 3

^1^ SH = synovial hypertrophy; ^2^ PD = power Doppler.

Tenosynovitis represents an inflammation of the synovial sheath of the tendons, which may lead to reduced joint mobility [42]. Ultrasound has a sensitivity of 86.5%, specificity of 100%, positive predictive value of 100%, negative predictive value of 92.3%, and an accuracy of 94.8% in identifying peritendinous inflammatory processes [43]. On ultrasound, tenosynovitis is characterized by peritendinous fluid accumulation and the presence of a Doppler signal within the synovial sheath [44].

Erosive arthritis was long considered a rare systemic manifestation of Sjögren’s syndrome, being more frequently attributed to rheumatoid arthritis [37]. The use of ultrasonography to evaluate structural changes in the small joints of the hand in patients with Sjögren’s syndrome allows the detection of bone erosions, which develop in the context of persistent intra-articular inflammation [39,45], with a higher prevalence. Ultrasonography has a sensitivity of 67.2%, specificity of 97.5%, positive predictive value of 84.8%, negative predictive value of 90.5%, and an accuracy of 91.5% for identifying cortical bone defects [43]. The coexistence of Sjögren’s syndrome increases the severity of erosive changes in rheumatoid arthritis [46].

The diagnostic performance of ultrasound in the evaluation of articular involvement in primary Sjögren’s syndrome is summarized in Table 6.

**Table 6:** Diagnostic performance of ultrasonography in evaluation of articular involvement in primary Sjögren’s syndrome [40,43].

Articular Involvement	Se ^4^ (%)	Sp ^5^ (%)
Synovitis		
RC ^1^ joint	73	78
MCP ^2^ joint	64	93
PIP ^3^ joint	71	94
Tenosynovitis	86.5	100
Erosive arthritis	67.2	97.5

^1^ RC = radiocarpal; ^2^ MCP = metacarpophalangeal; ^3^ PIP = proximal interphalangeal; ^4^ Se = sensitivity; ^5^ Sp = specificity.

### 3.3. Imaging Evaluation of Pulmonary Involvement in Sjögren’s Syndrome

Pulmonary involvement in patients with Sjögren’s syndrome manifests with symptoms such as persistent cough and dyspnea, associated with abnormal findings on diagnostic tests (pulmonary function tests and high-resolution computed tomography—HRCT) [37]. Pulmonary manifestations result from diffuse interstitial pathologies that develop in these patients, the most frequent histologic subtype being non-specific interstitial pneumonia (NSIP) [47], followed by usual interstitial pneumonia (UIP), lymphoid interstitial pneumonia (LIP), and organizing pneumonia (OP) [24]. The prevalence of interstitial lung disease among patients with Sjögren’s syndrome is 23% [24].

**Figure 3:**
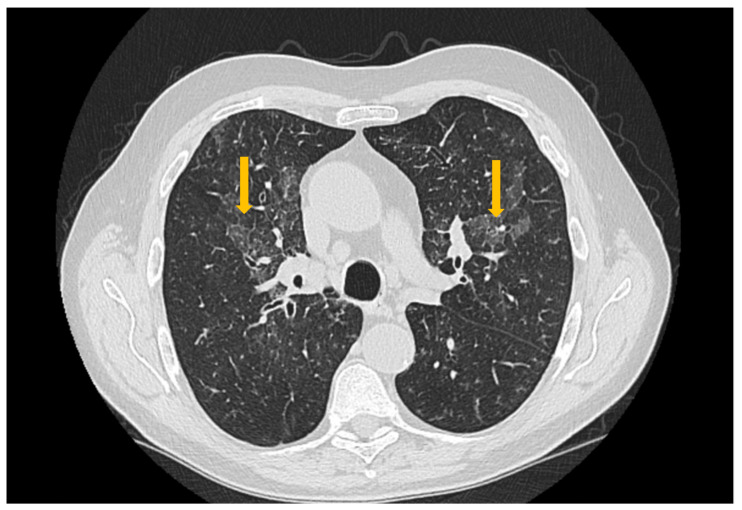
High-resolution computed tomography (HRCT) of the chest in non-specific interstitial pneumonia (NSIP). Axial HRCT image demonstrates bilateral, diffuse ground-glass opacities (arrows), associated with interlobular septal thickening and traction bronchiectasis, findings characteristic of fibrotic NSIP.

**Figure 4:**
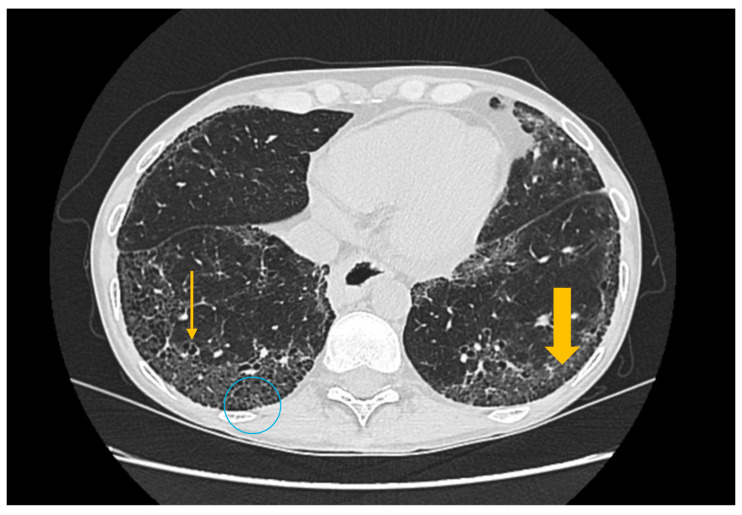
High-resolution computed tomography (HRCT) of the chest in usual interstitial pneumonia (UIP). Axial HRCT image demonstrates subpleural reticulations (thick arrow), traction bronchiectasis (thin arrow), and honeycombing (circle), with an asymmetric distribution and basal predominance, findings characteristic of UIP.

**Figure 5:**
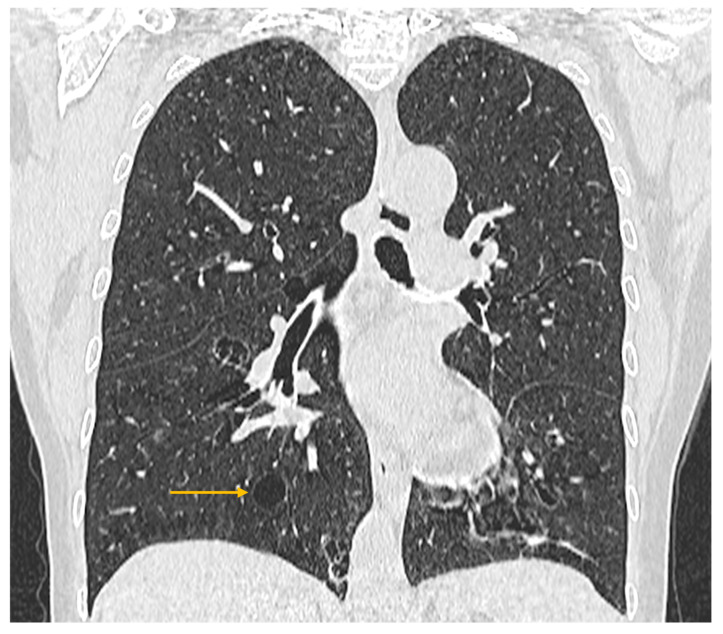
High-resolution computed tomography (HRCT) of the chest in lymphocytic interstitial pneumonia (LIP). Coronal HRCT image demonstrates thin-walled pulmonary cysts (arrow), a characteristic imaging feature of LIP.

High-resolution computed tomography represents the gold standard for evaluating pulmonary changes in diffuse interstitial lung diseases [48]. This imaging technique has a sensitivity of 100%, specificity of 82%, positive predictive value of 97%, and negative predictive value of 100% for the diagnosis of diffuse interstitial disease [49]. The imaging features vary according to the histologic subtype of involvement (Figure 3, Figure 4 and Figure 5) [50].

### 3.4. Imaging Evaluation of Central Nervous System Involvement in Sjögren’s Syndrome

Central nervous system involvement is reported in approximately 10.8% of patients with Sjögren’s syndrome, manifesting as headache, as well as sensory–motor syndromes and visual disturbances [25,51]. Magnetic resonance imaging plays an essential role in detecting structural changes within the central nervous system in the context of Sjögren’s syndrome [16,52].

Headache, usually of a migrainous character, is frequently associated with anti-SSA antibodies and Raynaud’s phenomenon. This association supports the hypothesis of vascular endothelial dysfunction or immune-mediated inflammatory injury affecting the cerebral microcirculation [51]. Imaging reveals ischemic lacunar lesions in the basal ganglia, without a statistically significant association with atherosclerotic plaques identified on color Doppler ultrasound [51].

**Figure 6:**
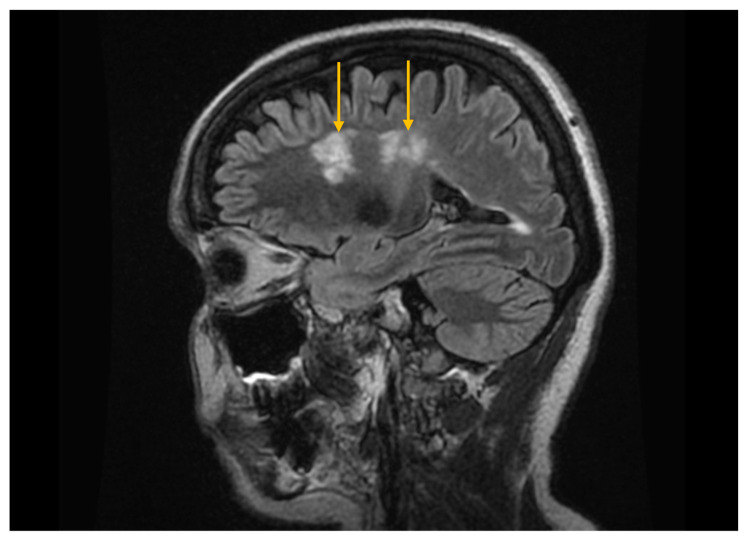
Magnetic resonance imaging (MRI) of the brain using the fluid-attenuated inversion recovery (FLAIR) sequence. Sagittal FLAIR MRI image demonstrates hyperintense cerebral demyelinating lesions (arrows), with a distribution pattern similar to that seen in multiple sclerosis.

In patients with Sjögren’s syndrome and neurological involvement, MRI may reveal cerebral demyelinating lesions with a distribution and appearance similar to those observed in multiple sclerosis (Figure 6) [25]. However, the published data regarding their significance are contradictory; some studies report a higher prevalence of demyelinating changes in Sjögren’s syndrome compared with control groups, while others do not demonstrate significant differences, suggesting that these lesions may instead reflect changes associated with normal physiological aging [53,54].

**Figure 7:**
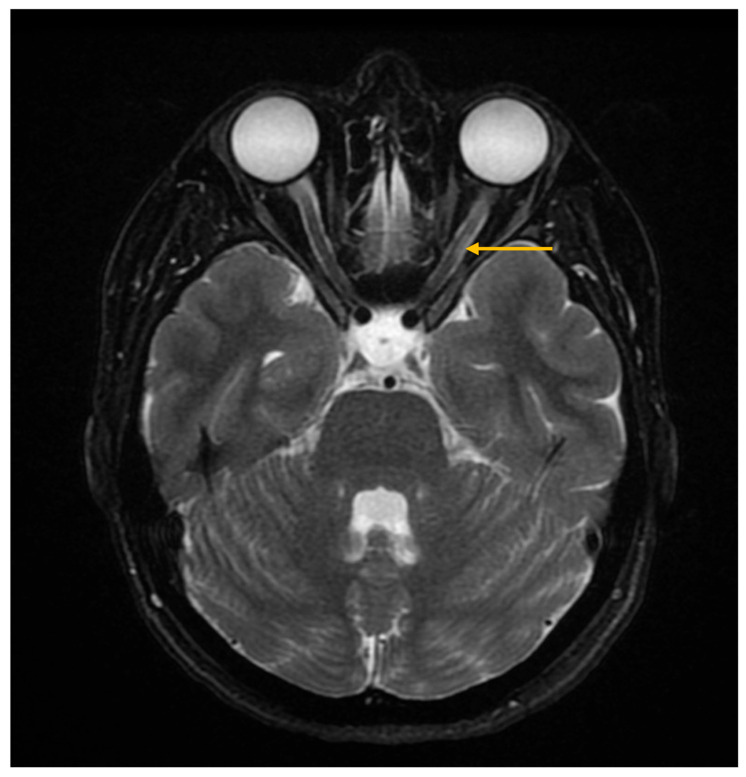
Magnetic resonance imaging (MRI) of the brain and orbits, axial T2-weighted short tau inversion recovery (STIR) sequence. The image demonstrates hyperintensity of the left optic nerve (arrow), consistent with optic neuritis.

In some patients with Sjögren’s syndrome and neurological manifestations, MRI may reveal lesions with features characteristic of neuromyelitis optica or disorders within the neuromyelitis optica spectrum, suggesting a possible association between the conditions (Figure 7) [16,25,52].

More rarely, pseudotumoral brain lesions may occur, characterized by mass effect, perilesional edema, absence of diffusion restriction, and peripheral contrast enhancement [55,56,57,58,59]. In some reported cases, histopathologic examination reveals changes consistent with gliosis or vasculitis, and treatment is based on corticosteroid therapy, which leads to improvement of focal symptoms and regression of the lesions on imaging [55,56].

### 3.5. Imaging Evaluation of Lymphoproliferative Complications in Sjögren’s Syndrome

B lymphocytes play a central role in the pathogenesis of Sjögren’s syndrome through a process of chronic activation that contributes to immune system dysfunction [10]. This sustained stimulation is reflected in the increased risk of developing non-Hodgkin lymphoma, particularly the mucosa-associated lymphoid tissue (MALT) subtype [60,61,62]. Modern imaging techniques—ultrasonography and magnetic resonance imaging—are valuable for monitoring patients with Sjögren’s syndrome to enable early detection of lymphoma [63].

**Figure 8:**
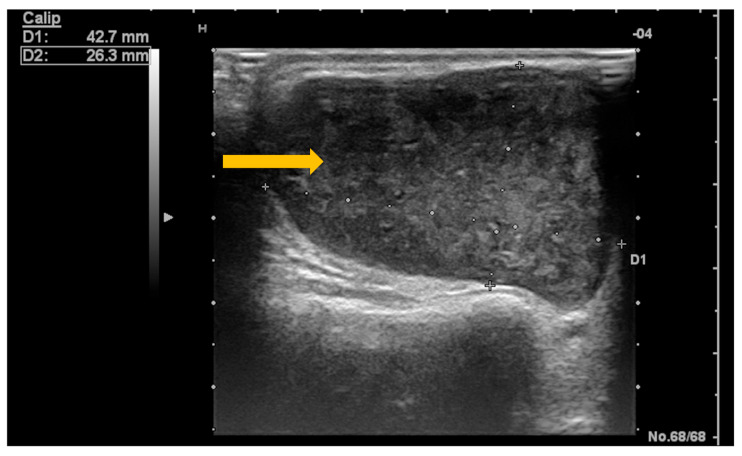
B-mode ultrasound of the parotid gland in a patient with Sjögren’s syndrome. Gray-scale (B-mode) ultrasound demonstrates a newly developed hypoechoic nodular lesion within the parotid gland (arrow), a finding suspicious for salivary gland lymphoma in the clinical context of Sjögren’s syndrome.

**Figure 9:**
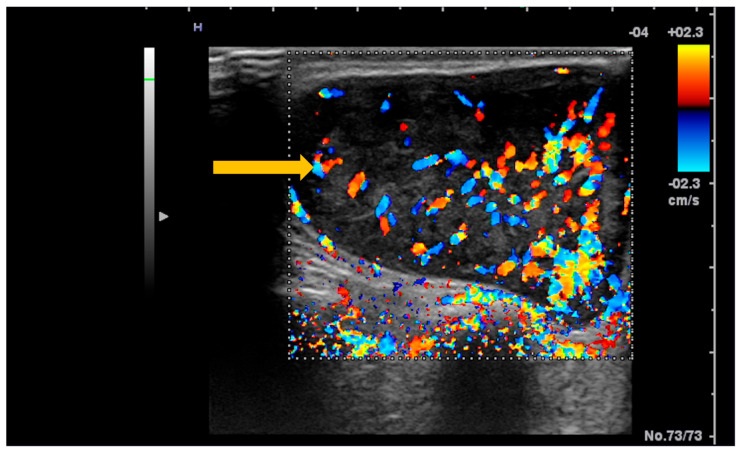
Color Doppler ultrasound of the parotid gland in a patient with Sjögren’s syndrome. Color Doppler imaging demonstrates a newly developed hypervascular nodular lesion within the parotid gland (arrow), a finding suspicious for salivary gland lymphoma in the clinical context of Sjögren’s syndrome.

Ultrasonography is the imaging tool most frequently used in clinical practice due to its non-invasive nature, low cost, and wide availability [64]. Dynamic ultrasonographic evaluation of the salivary glands can identify a newly developed hypervascular, oval, relatively well-defined, hypoechoic nodular lesion with intrinsic hyperechoic septa and posterior acoustic enhancement (Figure 8 and Figure 9) [64,65]. At the same time, ultrasonography can be used to guide biopsies of lesions with suspicious imaging characteristics [63].

**Figure 10:**
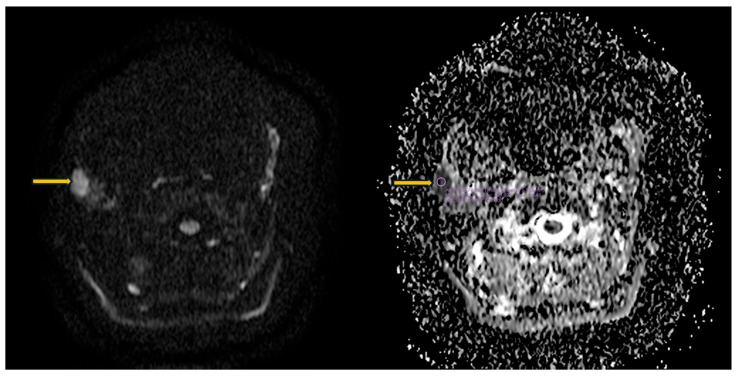
MRI diffusion-weighted imaging (DWI) and ADC map of the parotid gland. Axial images show a right parotid nodular lesion with restricted diffusion (arrows). The purple circle indicates the region of interest (ROI) used for calculation of the apparent diffusion coefficient (ADC), which is markedly reduced (0.460 × 10^−3^ mm^2^/s), a pattern suspicious for salivary gland lymphoma in the context of Sjögren’s syndrome.

Magnetic resonance imaging is frequently used for the diagnosis and local staging of lymphoma arising within the salivary glands in Sjögren’s syndrome. Owing to their increased cellularity, lymphomatous lesions show diffusion restriction, with low apparent diffusion coefficient (ADC) values (approximately 0.64 × 10^−3^ mm^2^/s) (Figure 10) [66]. On dynamic contrast-enhanced MRI, lymphoma typically demonstrates a type III enhancement curve, characterized by rapid gadolinium uptake and washout [67]. The time-to-peak (TTP) is the most sensitive parameter for distinguishing MALT lymphoma from benign lymphoepithelial lesions. Using a cut-off value of 79.65 s, TTP has an overall diagnostic accuracy of 94.7%, with a sensitivity of 94.1% and specificity of 95.2%. TTP values below this threshold are strongly associated with malignancy, whereas longer TTP values are indicative of benign lesions, reflecting differences in tumor vascularity and contrast kinetics [67].

In recent years, MRI radiomics (textural analysis on STIR/T2 and ADC sequences) has shown potential for risk stratification of lymphoma in patients with Sjögren’s syndrome and for differentiating benign from malignant parotid tumors, demonstrating high performance (AUC~0.931) in internal validation models [68], representing a promising direction for future research.

## 4. Conclusions

Modern imaging techniques play a central role in the multisystem evaluation of patients with Sjögren’s syndrome. Ultrasonography is a non-invasive, accessible, and reproducible imaging method, frequently used in clinical practice to identify and characterize changes within the muscular and articular systems, such as myopathies, synovitis, tenosynovitis, joint effusion, and bone erosions. High-resolution computed tomography accurately detects pulmonary abnormalities, particularly diffuse interstitial disease. Magnetic resonance imaging provides higher resolution than ultrasound and is valuable for diagnosing lymphoproliferative complications and assessing articular and neurological involvement.

Integrating these imaging modalities with clinical and paraclinical data enables a comprehensive approach to patients with Sjögren’s syndrome, facilitating early diagnosis, differentiation from other pathologies, monitoring disease progression, and detection of extraglandular complications arising in the context of immune dysregulation and persistent systemic inflammation.

## Data Availability

No new data were created or analyzed in this study. Data sharing is not applicable to this article.

## Abbreviations

The following abbreviations are used in this manuscript:

ACR/EULAR	American College of Rheumatology/European League Against Rheumatism
ADC	Apparent diffusion coefficient
Anti-SSA	anti-Sjögren’s syndrome-related antigen A
AUC	Area under the curve
CT	Computed tomography
ESSDAI	European League Against Rheumatism Sjögren’s Syndrome Disease Activity Index
FLAIR	Fluid-attenuated inversion recovery
HRCT	High-resolution computed tomography
IBM	Inclusion body myositis
LIP	Lymphoid interstitial pneumonia
MALT	Mucosa-associated lymphoid tissue
MCP	Metacarpophalangeal
MRI	Magnetic resonance imaging
NSIP	Non-specific interstitial pneumonia
OMERACT	Outcome Measures in Rheumatology
OP	Organizing pneumonia
PD	Power Doppler
PIP	Interphalangeal
RC	Radiocarpal
SE	Strain elastography
SH	Synovial hypertrophy
STIR	Short tau inversion recovery
SWE	Shear-wave elastography
UIP	Usual interstitial pneumonia
US	Ultrasonography

## References

[1] André F., Böckle B.C. (2022). Sjögren’s syndrome. J. Dtsch. Dermatol. Ges..

[2] Baldini C., Talarico R., Tzioufas A.G., Bombardieri S. (2025). Primary Sjogren Syndrome. StatPearls.

[3] Negrini S., Emmi G., Greco M., Borro M., Sardanelli F., Murdaca G., Indiveri F., Puppo F. (2022). Sjögren’s syndrome: A systemic autoimmune disease. Clin. Exp. Med..

[4] Maleki-Fischbach M., Kastsianok L., Koslow M., Chan E.D. (2024). Manifestations and management of Sjögren’s disease. Arthritis Res. Ther..

[5] Lodba A., Ancuta C., Tatarciuc D., Ghiorghe A., Lodba L.O., Iordache C. (2024). Comparative Analysis of Glandular and Extraglandular Manifestations in Primary and Secondary Sjögren’s Syndrome: A Study in Two Academic Centers in North-East Romania. Diagnostics.

[6] Zabotti A., Zandonella Callegher S., Tullio A., Vukicevic A., Hocevar A., Milic V., Cafaro G., Carotti M., Delli K., De Lucia O. (2020). Salivary Gland Ultrasonography in Sjögren’s Syndrome: A European Multicenter Reliability Exercise for the HarmonicSS Project. Front. Med..

[7] Shiboski C.H., Shiboski S.C., Seror R., Criswell L.A., Labetoulle M., Lietman T.M., Rasmussen A., Scofield H., Vitali C., Bowman S.J. (2017). 2016 American College of Rheumatology/European League Against Rheumatism Classification Criteria for Primary Sjögren’s Syndrome: A Consensus and Data-Driven Methodology Involving Three International Patient Cohorts. Arthritis Rheumatol..

[8] Horai Y., Shimizu T., Nakamura H., Kawakami A. (2024). Recent Advances in Pathogenesis, Diagnostic Imaging, and Treatment of Sjögren’s Syndrome. J. Clin. Med..

[9] Wang K.Y., Wintermark M., Penta M. (2022). Imaging characteristics of Sjögren’s syndrome. Clin. Imaging.

[10] Mihai A., Caruntu C., Jurcut C., Blajut F.C., Casian M., Opris-Belinski D., Ionescu R., Caruntu A. (2023). The Spectrum of Extraglandular Manifestations in Primary Sjögren’s Syndrome. J. Pers. Med..

[11] Hočevar A., Bruyn G.A., Terslev L., De Agustin J.J., MacCarter D., Chrysidis S., Collado P., Dejaco C., Fana V., Filippou G. (2022). Development of a new ultrasound scoring system to evaluate glandular inflammation in Sjögren’s syndrome: An OMERACT reliability exercise. Rheumatology.

[12] Martel A., Coiffier G., Bleuzen A., Goasguen J., de Bandt M., Deligny C., Magnant J., Ferreira N., Diot E., Perdriger A. (2019). What is the best salivary gland ultrasonography scoring methods for the diagnosis of primary or secondary Sjögren’s syndromes?. Jt. Bone Spine.

[13] Kojima I., Sakamoto M., Iikubo M., Kumamoto H., Muroi A., Sugawara Y., Satoh-Kuriwada S., Sasano T. (2017). Diagnostic performance of MR imaging of three major salivary glands for Sjögren’s syndrome. Oral Dis..

[14] Van Ginkel M.S., Glaudemans A.W.J.M., van der Vegt B., Mossel E., Kroese F.G.M., Bootsma H., Vissink A. (2020). Imaging in Primary Sjögren’s Syndrome. J. Clin. Med..

[15] Gupta S., Ferrada M.A., Hasni S.A. (2019). Pulmonary Manifestations of Primary Sjögren’s Syndrome: Underlying Immunological Mechanisms, Clinical Presentation, and Management. Front. Immunol..

[16] McCoy S.S., Baer A.N. (2017). Neurological Complications of Sjögren’s Syndrome: Diagnosis and Management. Curr. Treatm. Opt. Rheumatol..

[17] Tonami H., Matoba M., Kuginuki Y., Yokota H., Higashi K., Yamamoto I., Sugai S. (2003). Clinical and imaging findings of lymphoma in patients with Sjögren syndrome. J. Comput. Assist. Tomogr..

[18] Flores-Chávez A., Kostov B., Solans R., Fraile G., Maure B., Feijoo-Massó C., Rascón F.J., Pérez Álvarez R., Zamora-Pasadas M., García-Pérez A. (2018). Severe, life-threatening phenotype of primary Sjögren’s syndrome: Clinical characterisation and outcomes in 1580 patients (GEAS-SS Registry). Clin. Exp. Rheumatol..

[19] Seror R., Gottenberg J.E., Devauchelle-Pensec V., Dubost J.J., Le Guern V., Hayem G., Fauchais A., Goeb V., Hachulla E., Hatron P.Y. (2013). European League Against Rheumatism Sjögren’s Syndrome Disease Activity Index and European League Against Rheumatism Sjögren’s Syndrome Patient-Reported Index: A Complete Picture of Primary Sjögren’s Syndrome Patient. Arthritis Care Res..

[20] Seror R., Ravaud P., Bowman S.J., Baron G., Tzioufas A., Theander E., Gottenberg J.-E., Bootsma H., Mariette X., Vitali C. (2010). EULAR Sjogren’s syndrome disease activity index: Development of a consensus systemic disease activity index for primary Sjogren’s syndrome. Ann. Rheum. Dis..

[21] Felten R., Giannini M., Nespola B., Lannes B., Levy D., Seror R., Vittecoq O., Hachulla E., Perdriger A., Dieude P. (2021). Refining myositis associated with primary Sjögren’s syndrome: Data from the prospective cohort ASSESS. Rheumatology.

[22] Colafrancesco S., Priori R., Gattamelata A., Picarelli G., Minniti A., Brancatisano F., D’Amati G., Giordano C., Cerbelli B., Maset M. (2015). Myositis in primary Sjögren’s syndrome: Data from a multicentre cohort. Clin. Exp. Rheumatol..

[23] Ramos-Casals M., Brito-Zerón P., Solans R., Camps M.T., Casanovas A., Sopeña B., Diaz-Lopez B., Rascon F.-J., Qanneta R., Fraile G. (2014). Systemic involvement in primary Sjogren’s syndrome evaluated by the EULAR-SS disease activity index: Analysis of 921 Spanish patients (GEAS-SS Registry). Rheumatology.

[24] Berardicurti O., Marino A., Genovali I., Navarini L., D’Andrea S., Currado D., Rigon A., Arcarese L., Vadacca M., Giacomelli R. (2023). Interstitial Lung Disease and Pulmonary Damage in Primary Sjögren’s Syndrome: A Systematic Review and Meta-Analysis. J. Clin. Med..

[25] Afzali A.M., Moog P., Kalluri S.R., Hofauer B., Knopf A., Kirschke J.S., Hemmer B., Berthele A. (2023). CNS demyelinating events in primary Sjögren’s syndrome: A single-center case series on the clinical phenotype. Front. Neurol..

[26] Konen F.F., Güzeloglu Y.E., Seeliger T., Jendretzky K.F., Nay S., Grote-Levi L., Schwenkenbecher P., Gründges C., Ernst D., Witte T. (2025). Idiopathic inflammatory myopathy associated with Sjögren’s disease: Features of a distinct clinical entity. Front. Immunol..

[27] Espitia-Thibault A., Masseau A., Néel A., Espitia O., Toquet C., Mussini J.M., Hamidou M. (2017). Sjögren’s syndrome-associated myositis with germinal centre-like structures. Autoimmun. Rev..

[28] Giannini M., Felten R., Gottenberg J.E., Geny B., Meyer A. (2022). Inclusion body myositis and Sjögren’s syndrome: The association works both ways. Acta Neuropathol. Commun..

[29] Leeuwenberg K.E., van Alfen N., Christopher-Stine L., Paik J.J., Tiniakou E., Mecoli C., Doorduin J., Saris C.G., Albayda J. (2020). Ultrasound can differentiate inclusion body myositis from disease mimics. Muscle Nerve.

[30] Tasca G., Monforte M., De Fino C., Kley R.A., Ricci E., Mirabella M. (2015). Magnetic resonance imaging pattern recognition in sporadic inclusion-body myositis. Muscle Nerve.

[31] De Visser M., Carlier P., Vencovský J., Kubínová K., Preusse C., Albayda J., Allenbach Y., Benveniste O., Diederichsen L., Demonceau G. (2023). 255th ENMC workshop: Muscle imaging in idiopathic inflammatory myopathies. 15th January, 16th January and 22nd January 2021—Virtual meeting and hybrid meeting on 9th and 19th September 2022 in Hoofddorp, The Netherlands. Neuromuscul. Disord..

[32] Albayda J., van Alfen N. (2020). Diagnostic Value of Muscle Ultrasound for Myopathies and Myositis. Curr. Rheumatol. Rep..

[33] Abdelnaby R., Mohamed K.A., Elgenidy A., Sonbol Y.T., Bedewy M.M., Aboutaleb A.M., Ebrahim M.A., Maallem I., Dardeer K.T., Heikal H.A. (2022). Muscle Sonography in Inclusion Body Myositis: A Systematic Review and Meta-Analysis of 944 Measurements. Cells.

[34] Heckmatt J.Z., Leeman S., Dubowitz V. (1982). Ultrasound imaging in the diagnosis of muscle disease. J. Pediatr..

[35] Botar-Jid C., Damian L., Dudea S.M., Vasilescu D., Rednic S., Badea R. (2010). The contribution of ultrasonography and sonoelastography in assessment of myositis. Med. Ultrason..

[36] Alfuraih A.M., O’Connor P., Tan A.L., Hensor E.M.A., Ladas A., Emery P., Wakefield R.J. (2019). Muscle shear wave elastography in idiopathic inflammatory myopathies: A case-control study with MRI correlation. Skelet. Radiol..

[37] Ramos-Casals M., Brito-Zerón P., Seror R., Bootsma H., Bowman S.J., Dörner T., Gottenberg J.-E., Mariette X., Theander E., Bombardieri S. (2015). Characterization of systemic disease in primary Sjögren’s syndrome: EULAR-SS Task Force recommendations for articular, cutaneous, pulmonary and renal involvements. Rheumatology.

[38] Carubbi F., Alunno A., Conforti A., Riccucci I., Di Cola I., Bartoloni E., Gerli R. (2020). Characterisation of articular manifestations in primary Sjögren’s syndrome: Clinical and imaging features. Clin. Exp. Rheumatol..

[39] Fujimura T., Fujimoto T., Hara R., Shimmyo N., Kobata Y., Kido A., Akai Y., Tanaka Y. (2015). Subclinical articular involvement in primary Sjögren’s syndrome assessed by ultrasonography and its negative association with anti-centromere antibody. Mod. Rheumatol..

[40] Takase-Minegishi K., Horita N., Kobayashi K., Yoshimi R., Kirino Y., Ohno S., Kaneko T., Nakajima H., Wakefield R.J., Emery P. (2018). Diagnostic test accuracy of ultrasound for synovitis in rheumatoid arthritis: Systematic review and meta-analysis. Rheumatology.

[41] D’Agostino M.A., Terslev L., Aegerter P., Backhaus M., Balint P., Bruyn G.A., Filippucci E., Grassi W., Iagnocco A., Jousse-Joulin S. (2017). Scoring ultrasound synovitis in rheumatoid arthritis: A EULAR-OMERACT ultrasound taskforce-Part 1: Definition and development of a standardised, consensus-based scoring system. RMD Open.

[42] Erol K., Akyildiz Tezcan E., Akgöl S. (2025). Exploring hand function in newly diagnosed primary Sjögren’s syndrome: Clinical, radiographic, and ultrasonographic insights. J. Hand Ther..

[43] Malla S., Vyas S., Bhalla A.S., Kumar U., Kumar S., Gupta A.K. (2020). Ultrasonography in Early Rheumatoid Arthritis of Hand and Wrist Joints: Comparison with Magnetic Resonance Imaging. Indian J. Orthop..

[44] Guedes L.K.N., Leon E.P., Bocate T.S., Bonfigliolli K.R., Lourenço S.V., Bonfa E., Pasoto S.G. (2020). Characterizing hand and wrist ultrasound pattern in primary Sjögren’s syndrome: A case-control study. Clin. Rheumatol..

[45] Boutry N., Hachulla E., Flipo R.M., Cortet B., Cotten A. (2005). MR imaging findings in hands in early rheumatoid arthritis: Comparison with those in systemic lupus erythematosus and primary Sjögren syndrome. Radiology.

[46] Brown L.E., Frits M.L., Iannaccone C.K., Weinblatt M.E., Shadick N.A., Liao K.P. (2015). Clinical characteristics of RA patients with secondary SS and association with joint damage. Rheumatology.

[47] Ito I., Nagai S., Kitaichi M., Nicholson A.G., Johkoh T., Noma S., Kim D.S., Handa T., Izumi T., Mishima M. (2005). Pulmonary manifestations of primary Sjogren’s syndrome: A clinical, radiologic, and pathologic study. Am. J. Respir. Crit. Care Med..

[48] Drimus J.C., Duma R.C., Trăilă D., Mogoșan C.D., Manolescu D.L., Fira-Mladinescu O. (2025). High-Resolution CT Findings in Interstitial Lung Disease Associated with Connective Tissue Diseases: Differentiating Patterns for Clinical Practice-A Systematic Review with Meta-Analysis. J. Clin. Med..

[49] Yan J.H., Pan L., Gao Y.B., Cui G.H., Wang Y.H. (2021). Utility of lung ultrasound to identify interstitial lung disease: An observational study based on the STROBE guidelines. Medicine.

[50] La Rocca G., Ferro F., Sambataro G., Elefante E., Fonzetti S., Fulvio G., Navarro I.C., Mosca M., Baldini C. (2023). Primary-Sjögren’s-Syndrome-Related Interstitial Lung Disease: A Clinical Review Discussing Current Controversies. J. Clin. Med..

[51] Khalayli N., Bouri M.F., Wahbeh M., Drie T., Kudsi M. (2023). Neurological injury in primary Sjogren’s syndrome. Ann. Med. Surg..

[52] Módis L.V., Aradi Z., Horváth I.F., Bencze J., Papp T., Emri M., Berényi E., Bugán A., Szántó A. (2022). Central Nervous System Involvement in Primary Sjögren’s Syndrome: Narrative Review of MRI Findings. Diagnostics.

[53] Tzarouchi L.C., Tsifetaki N., Konitsiotis S., Zikou A., Astrakas L., Drosos A., Argyropoulou M.I. (2011). CNS involvement in primary Sjogren Syndrome: Assessment of gray and white matter changes with MRI and voxel-based morphometry. AJR Am. J. Roentgenol..

[54] Harboe E., Beyer M.K., Greve O.J., Gøransson L.G., Tjensvoll A.B., Kvaløy J.T., Omdal R. (2009). Cerebral white matter hyperintensities are not increased in patients with primary Sjögren’s syndrome. Eur. J. Neurol..

[55] Corrêa D.G., da Hygino da Cruz L.C., Dos Santos R.Q., Marcondes J., de Abreu M.M. (2024). Brain tumefactive vasculitis in primary Sjögren syndrome. Int. J. Rheum. Dis..

[56] Sassi S.B., Nabli F., Boubaker A., Ghorbel I.B., Neji S., Hentati F. (2014). Pseudotumoral brain lesion as the presenting feature of primary Sjögren’s syndrome. J. Neurol. Sci..

[57] Michel L., Toulgoat F., Desal H., Laplaud D.A., Magot A., Hamidou M., Wiertlewski S. (2011). Atypical neurologic complications in patients with primary Sjögren’s syndrome: Report of 4 cases. Semin. Arthritis Rheum..

[58] Sanahuja J., Ordoñez-Palau S., Begué R., Brieva L., Boquet D. (2008). Primary Sjögren Syndrome with tumefactive central nervous system involvement. AJNR Am. J. Neuroradiol..

[59] Niu B., Zou Z., Shen Y., Cao B. (2017). A case report of Sjögren syndrome manifesting bilateral basal ganglia lesions. Medicine.

[60] Stergiou I.E., Chatzis L.G., Pezoulas V.C., Baldini C., Fotiadis D.I., Voulgarelis M., Tzioufas A.G., Goules A.V. (2022). The clinical phenotype of primary Sjögren’s syndrome patients with lymphadenopathy. Clin. Exp. Rheumatol..

[61] Rodolfi S., Della-Torre E., Bongiovanni L., Mehta P., Fajgenbaum D.C., Selmi C. (2024). Lymphadenopathy in the rheumatology practice: A pragmatic approach. Rheumatology.

[62] Seror R., Bowman S.J., Brito-Zeron P., Theander E., Bootsma H., Tzioufas A., Gottenberg J.-E., Ramos-Casals M., Dörner T., Ravaud P. (2015). EULAR Sjögren’s syndrome disease activity index (ESSDAI): A user guide. RMD Open.

[63] Vissink A., van Ginkel M.S., Bootsma H., Glaudemans A., Delli K. (2024). At the cutting-edge: What’s the latest in imaging to diagnose Sjögren’s disease?. Expert Rev. Clin. Immunol..

[64] Yang J., Park Y., Lee J.J., Kim W.U., Park S.H., Kwok S.K. (2025). Clinical value of salivary gland ultrasonography in evaluating secretory function, disease activity, and lymphoma risk factors in primary Sjögren’s syndrome. Clin. Rheumatol..

[65] Lorenzon M., Spina E., Tulipano Di Franco F., Giovannini I., De Vita S., Zabotti A. (2022). Salivary Gland Ultrasound in Primary Sjögren’s Syndrome: Current and Future Perspectives. Open Access Rheumatol..

[66] Kato H., Kanematsu M., Goto H., Mizuta K., Aoki M., Kuze B., Hirose Y. (2012). Mucosa-associated lymphoid tissue lymphoma of the salivary glands: MR imaging findings including diffusion-weighted imaging. Eur. J. Radiol..

[67] Zhu L., Zhang C., Hua Y., Yang J., Yu Q., Tao X., Zheng J. (2016). Dynamic contrast-enhanced MR in the diagnosis of lympho-associated benign and malignant lesions in the parotid gland. Dentomaxillofac. Radiol..

[68] Muntean D.D., Lenghel L.M., Ștefan P.A., Fodor D., Bădărînză M., Csutak C., Dudea S.M., Rusu G.M. (2023). Radiomic Features Associated with Lymphoma Development in the Parotid Glands of Patients with Primary Sjögren’s Syndrome. Cancers.

